# VRK1 Depletion Facilitates the Synthetic Lethality of Temozolomide and Olaparib in Glioblastoma Cells

**DOI:** 10.3389/fcell.2021.683038

**Published:** 2021-06-14

**Authors:** Elena Navarro-Carrasco, Pedro A. Lazo

**Affiliations:** ^1^Molecular Mechanisms of Cancer Program, Instituto de Biología Molecular y Celular del Cáncer, Consejo Superior de Investigaciones Científicas (CSIC)-Universidad de Salamanca, Salamanca, Spain; ^2^Instituto de Investigación Biomédica de Salamanca-IBSAL, Hospital Universitario de Salamanca, Salamanca, Spain

**Keywords:** glioblastoma, temozolomide, olaparib, VRK1, DNA damage response

## Abstract

**Background:**

Glioblastomas treated with temozolomide frequently develop resistance to pharmacological treatments. Therefore, there is a need to find alternative drug targets to reduce treatment resistance based on tumor dependencies. A possibility is to target simultaneously two proteins from different DNA-damage repair pathways to facilitate tumor cell death. Therefore, we tested whether targeting the human chromatin kinase VRK1 by RNA interference can identify this protein as a novel molecular target to reduce the dependence on temozolomide in combination with olaparib, based on synthetic lethality.

**Materials and Methods:**

Depletion of VRK1, an enzyme that regulates chromatin dynamic reorganization and facilitates resistance to DNA damage, was performed in glioblastoma cells treated with temozolomide, an alkylating agent used for GBM treatment; and olaparib, an inhibitor of PARP-1, used as sensitizer. Two genetically different human glioblastoma cell lines, LN-18 and LN-229, were used for these experiments. The effect on the DNA-damage response was followed by determination of sequential steps in this process: H4K16ac, γH2AX, H4K20me2, and 53BP1.

**Results:**

The combination of temozolomide and olaparib increased DNA damage detected by labeling free DNA ends, and chromatin relaxation detected by H4K16ac. The combination of both drugs, at lower doses, resulted in an increase in the DNA damage response detected by the formation of γH2AX and 53BP1 foci. VRK1 depletion did not prevent the generation of DNA damage in TUNEL assays, but significantly impaired the DNA damage response induced by temozolomide and olaparib, and mediated by γH2AX, H4K20me2, and 53BP1. The combination of these drugs in VRK1 depleted cells resulted in an increase of glioblastoma cell death detected by annexin V and the processing of PARP-1 and caspase-3.

**Conclusion:**

Depletion of the chromatin kinase VRK1 promotes tumor cell death at lower doses of a combination of temozolomide and olaparib treatments, and can be a novel alternative target for therapies based on synthetic lethality.

## Background

Glioblastomas (GBM) are a group of brain tumors with poor prognosis and limited therapeutic options. The current treatment for GBM is based on the use of temozolomide (TMZ) (Lee, 2016; Kaina and Christmann, 2019), in combination with radiotherapy and surgical resection (Perazzoli et al., 2015; Lee, 2016). TMZ is an alkylating agent that modifies DNA (Daniel et al., 2019) causing 06-meG, N3-meG, N7-meG, and N3-meA lesions. GBM cells have a high DNA damage response (DDR), which allows them to repair the lesions caused by TMZ. Resistance to TMZ occurs in GBM cells expressing high levels of MGMT (O-6-methylguanine-DNA methyltransferase) (Zhang et al., 2012), an enzyme that directly removes the methyl group added by TMZ to the O6-mG (Lee, 2016). Moreover, N7-meG, N3-meG, and N3-meA alkylating modifications are repaired by the Base Excision Repair pathway (BER) (Kennedy et al., 2018; Visnes et al., 2018; Kaina and Christmann, 2019; Mullins et al., 2019), which is initiated in a locally altered chromatin (Kennedy et al., 2018). BER requires the participation of poly (ADP-ribose) polymerase (PARP) (Ray Chaudhuri and Nussenzweig, 2017; Eisemann and Pascal, 2020), which is also a therapeutic target (Visnes et al., 2018). This process requires changes in chromatin relaxation (Ball and Yokomori, 2011; Cann and Dellaire, 2011), which is associated to histone acetylation mediated by KAT5/Tip60 acetyltransferase (Murr et al., 2006), that is regulated by VRK1 (Garcia-Gonzalez et al., 2020). Within a tumor, each cell has to respond to DNA damage independently of its individual situation regarding cell cycle phase, differentiation stage, local cell interactions and its microenvironment.

Drugs targeting PARP-1, such as olaparib, impair the DDR (Lord and Ashworth, 2017), and sensitize tumor cells to other treatments that cause DNA damage (Ray Chaudhuri and Nussenzweig, 2017), such as radiation therapy (McMahon et al., 2016). Olaparib targets BER pathway, which repairs alkylating lesions in the DNA (Ray Chaudhuri and Nussenzweig, 2017). The combined targeting of alternative pathways can be a suitable strategy to improve cancer management and treatment. Targeting participants in different DDR pathways is a therapeutic option in cancer treatment (O’Connor, 2015; Brown et al., 2017), which can be exploited in synthetic lethality strategies (McDonald et al., 2017; Sizemore et al., 2018), either genetic or pharmacologic (Chan et al., 2019; Kategaya et al., 2019; Lieb et al., 2019; McDermott et al., 2019). In this context, it is becoming a useful approach to target different pathways associated with DDR, in the form of synthetic lethality, and which can include taking advantage of mutations in DDR pathways in the tumor. Olaparib is used for the treatment of different types of cancer, including glioblastomas (Higuchi et al., 2020). Olaparib is frequently used to treat tumors that already have alterations in some DDR pathway, such as BRCA1 (Fong et al., 2009; Tewari et al., 2015), BRCA2 (Lohse et al., 2016), or WRN mutations (Kategaya et al., 2019). Synthetic lethality strategies imply the simultaneous targeting with drugs that impair different DNA repair pathways, or the combination of targeting a DDR pathway different from those that already have a mutation in one of them, such as in breast (Kaelin, 2005; Fong et al., 2009) and ovarian cancer (Tewari et al., 2015). Furthermore, tumors that respond exceptionally well to treatment often have mutations in genes associated to DNA repair (Wheeler et al., 2021). For this reason, we studied whether the antitumor effect of TMZ, in the context of DNA damage and DDR, could be enhanced by its combination with other drugs that target DDR proteins, such as olaparib (Higuchi et al., 2020). Alternatively, the interference with the chromatin kinase VRK1 (Campillo-Marcos and Lazo, 2019; Campillo-Marcos et al., 2021), could be a novel alternative strategy of synthetic lethality in glioblastomas.

Dynamic chromatin remodeling plays a critical role in sensitivity to DNA damage, which is higher in situations of relaxation such as transcription or replication (Rodriguez et al., 2015; Hauer and Gasser, 2017). Therefore, a chromatin kinase is likely to participate in this response. In this context, the nuclear chromatin kinase VRK1 regulates chromatin relaxation (Salzano et al., 2015; Monsalve et al., 2016) and could be a suitable candidate to play a role in this process (Campillo-Marcos and Lazo, 2018, 2019), since it is the most abundant nuclear kinase (Shiio et al., 2003; Varjosalo et al., 2013). On chromatin, VRK1 directly interacts with histones (Moura et al., 2018), affects chromatin reorganization and participates in specific steps in DNA damage response (Sanz-Garcia et al., 2012; Salzano et al., 2015; Monsalve et al., 2016). The kinase-activity of VRK1 is enhanced independently of the type of DNA damage (Sanz-Garcia et al., 2012). VRK1 is highly expressed in many tumor types (Martin et al., 2008) and confers resistance to genotoxic treatments in different tumors (Peters et al., 2005; Salzano et al., 2014; Liu et al., 2017; Jarman et al., 2018). Furthermore, VRK1 is associated to tumor cell proliferation (Valbuena et al., 2011), including neuroblastoma cells (Colmenero-Repiso et al., 2020). Overexpression of VRK1 is a marker of recurrence and promote temozolomide resistance in GBM (Varghese et al., 2016) and is a marker of dependency (Weinstein and Joe, 2006). Several CRISPR-enabled functional genomic screenings searching for new protein targets have identified VRK1 as a potential new therapeutic target in the context of cancer dependencies (Tiedemann et al., 2012; Kiessling et al., 2016; McDonald et al., 2017). Furthermore, VRK1 has an atypical kinase domain that can facilitate the development of specific inhibitors (Manning et al., 2002; Fedorov et al., 2007a; Counago et al., 2017). Synthetic lethality studies for drug discovery also detected that VRK1 is a potential target for this therapeutic approach (Huang et al., 2020). These characteristics make VRK1, for which no drugs are currently available, a suitable new target for its combination with other pharmacological treatments in order to facilitate the elimination of tumor cells. Combination of the functional inactivation of proteins involved in DDR, by mutations, or by treatment with specific drug, are the base of synthetic lethality. In this context, the activity of a chromatin kinase, such as VRK1, might be a novel alternative target once specific inhibitors are developed.

## Materials and Methods

### Glioblastoma Cell Lines

Two glioblastoma cell lines were used in this work. LN-18 (ATCC, CRL-2610, *TP53* mutated, and deletion of p16 and p14ARF), LN-229 (ATCC, CRL2611, *TP53* mutated, deletion of p16 and p14ARF, and MGMT deficient) (Fenstermaker et al., 1998; He and Kaina, 2019). These cell lines were grown in glutamine-free DMEM from SIGMA-ALDRICH supplemented with penicillin (50 U/ml) and streptomycin (50 μg/ml) (GIBCO-Life technologies), 10% FBS (fetal bovine serum) and L-glutamine (2 mM) cultivated in an incubator with fixed conditions: 5% CO_2_, 85–95% humidity and 37°C in flasks (BD Falcon; Rodriguez-Hernandez et al., 2013). Cells were detached with trypsin-EDTA (TryplETM, Thermo Fisher Scientific).

### VRK1 Depletion

Two different siRNA were used to deplete human VRK1. siVRK1-02 (siV1-02: CAAGGAACCTGGTGTTGAA) and siVRK1-03 (siV1-03: GGAAUGGAAAGUAGGAUUA). The “ON-TARGETplus siControl non-targeting siRNA” was used as negative control. All RNAi were from GE-Healthcare-Dharmacon. Opti-MEM (GIBCO-life technologies) was used for lipotransfectin and RNA dilution. RNA was used at 200 nM. The mix lipotransfectin-optiMEM-RNA was incubated for 30 min and added to the cells. Cells were incubated with antibiotic-free medium. The VRK1 siRNA used in this work are highly specific, and their effects are rescued by kinase-active VRK1 (human or murine), but they are not rescued by kinase-dead VRK1 (Sanz-Garcia et al., 2012; Cantarero et al., 2015; Salzano et al., 2015; Monsalve et al., 2016; Martin-Doncel et al., 2019; Marcos et al., 2020). Furthermore, kinase-dead VRK1 (K179E) also does not rescue the effects in response to DDR (Sanz-Garcia et al., 2012; Cantarero et al., 2015; Salzano et al., 2015; Monsalve et al., 2016; Martin-Doncel et al., 2019; Marcos et al., 2020).

### Immunofluorescence and Confocal Microscopy

Immunofluorescence (IF) assays were used to detect endogenous and/or transfected proteins in cell lines. Cell lines were cultured in glass coverslips (Thermo Scientific) in culture dishes. Cells were fixed with 3% paraformaldehyde (PFA) in PBS for 25 min at RT, and 200 mM glycine was added. Cells were permeabilized with 0.2% triton X-100 for 20 min. Later, cells were blocked with 1% BSA in PBS with 0.1% sodium azide for 1 h at RT, or overnight at 4°C. The first primary antibody was incubated between 2 and 4 h at room temperature or overnight at 4°C. Coverslips were washed 3 times with PBS and the second primary antibody was incubated between 2 and 4 h RT. Afterward, cells were washed with PBS and incubated with the secondary antibodies (Table 1) at 1:1,000 dilution, in the dark, for 1 h at RT, and finally washed 3 times with PBS in darkness. Nuclei were stained with DAPI (4’, 6-diamidino-2-phenylindole) at 1:1,000 dilution for 15 min in the dark, followed by three washes with PBS. Coverslips were mounted on microscope slides with MOWIOL 4-88 (Calbiochem; Billerica, MA, United States). Finally, cells were visualized using a Leica TCS SP5 inverted fluorescence confocal microscope (Leica Microsystems; Wetzlar, Germany) connected to a digital video camera Leica DC100 (Leica Microsystems). Image analysis was performed using ImageJ software.

**TABLE 1:** Antibodies used.

Primary antibodies				
Antibody	Origin	Dilution (WB/IF)	Clone, reference	Manufacturer
β-actin	Mouse monoclonal	1:2,000/-	AC15, A5441	Sigma-Aldrich
53BP1	Mouse monoclonal	-/1:400	NB100-304	Novus Biologicals
γH2AX	Mouse monoclonal	1:1,000/1:200	JBW301, 05-636	Millipore
VRK1	Mouse monoclonal	1:1,000/1:200	1B5	Valbuena et al., 2007
VRK1	Rabbit polyclonal	1:1,000/-	VC	Valbuena et al., 2007
VRK1 (N-term)	Rabbit polyclonal	1:1,000/1:200	HPA000660	Sigma-Aldrich
H4K16ac	Rabbit monoclonal	1:1,000/1:400/-	Ab109463	Abcam
H4K20me2	Rabbit polyclonal	1:500/1:400	9759	Cell Signaling
PARP1	Mouse monoclonal	1:1,000/-	Sc-8007	Santa Cruz
Caspase-3	Mouse monoclonal	1:1,000/-	Sc-7272	Santa Cruz
Cleaved caspase-3	Rabbit monoclonal	1:1,000-/-	9,664	Cell Signaling
**Secondary antibodies**				

**Antibody**	**Fluorochrome**	**Use, dilution**	**Reference**	**Manufacturer**

Goat anti-mouse IgG	Cy3 (Red)	IF, 1:1,000	115-165-146	Jackson Immunoresearch
Goat anti-rabbit IgG	Cy2 (Green)	IF, 1:1,000	111-225-144	Jackson Immunoresearch
Goat anti-mouse IgG	DyLight 680 (Red)	WB, 1:10,000	35518	Thermo Fisher Scientific

### TUNEL Assay

TUNEL assay (TdT-mediated dUTP Nick-End Labeling) (Roche) was used to label fragmented DNA in cells. Fluorescein-12-dUTP binds to the 3’-OH of the DNA strand and detected by a fluorescence microscope. Cells were cultured in glass coverslips and fixed in 3% PFA in PBS for 25 min at RT. PFA was removed, and 200 mM glycine was added for 15 min. Then, cells were permeabilized with 0.2% triton X-100 for 20 min. After that, cells were blocked with 1% BSA in PBS with 0.1% sodium azide for 1 h at RT, or overnight at 4°C. 50 μl of TUNEL reaction mixture was added (prepared according to the manufacturer). Coverslips were incubated for 1 h at 37°C in darkness. Coverslips were washed 3 times with PBS. Nuclei were stained with DAPI (4’, 6-diamidino-2-phenylindole), which was added at 1:1,000 dilution for 15 min in the dark. Finally, coverslips were washed 3 times with PBS, and mounted on microscope slides with MOWIOL 4-88 (Calbiochem, MA, United States). Samples were visualized using a Leica TCS SP5 inverted fluorescence confocal microscope and analyzed by ImageJ software.

### Protein Extraction and Quantification

All steps of protein extraction were carried out at 4°C. Cells were lysed using lysis buffer (50 mM Tris-HCl, pH 8.0, 150 mM NaCl, 1% Triton X-100, and 1 mM EDTA) supplemented with protease inhibitors (1 mM PMSF, aprotinin 10 μg/ml, and leupeptin 10 μg/ml) and phosphatase inhibitors (1 mM sodium orthovanadate and 1 mM sodium fluoride). Soft lysis buffer was added to the dishes and cells were scraped and transferred to a tube. Lysates were incubated for 20 min in ice, and centrifuged at 16,000 × g for 20 min. The pellet was discarded and the soluble fraction was kept and stored at −20°C. Protein extracts were quantified by Bradford assay (Bio-Rad). Samples with a known concentration of bovine serum albumin (BSA) from Bio-Rad were used for the standard curve. Samples were always prepared in duplicates. Absorbance was measured at 595 nm in a spectrophotometer (Bio-Rad).

### SDS-PAGE Electrophoresis

Three different acrylamide-bisacrylamide percentages for running gels were used depending on the size of the target protein. 12.5% gels were used for small proteins (<30 kDa), 10% gels for proteins between 30 and 100 kDa and 7.5% gels for proteins larger than 100 kDa. The running gel is composed of 7.5–12.5% acrylamide, 0.13–0.4% bis-acrylamide, in 0.375 M Tris-HCl (pH 8.8) and 3.5 mM SDS, tetramethylethylenediamine (TEMED) and ammonium persulfate (APS). The stacking gel added on top of the running gel is made of 4.8% acrylamide, 0.128% bis-acrylamide in 0.125 M Tris-HCl (pH 6.8) and 3.5 mM SDS, TEMED and APS. Protein extracts were mixed with the sample buffer (62.5 mM Tris-HCl (pH 6.8), 10% glycerol, 2.3% SDS, 0.1% bromophenol blue, and 5% β-mercaptoethanol), and the mix was boiled at 100°C for 5 min to denaturalize the proteins for gel loading. The electrophoresis was performed under denaturing conditions in electrophoresis buffer (25 mM Tris-HCl, 200 mM glycine, and 1.7 mM SDS). Precision plus Protein Standards Dual Color (Bio-Rad) was used as protein size markers.

### *In vitro* Kinase Assay

Endogenous VRK1 was immunoprecipitated as previously reported. The *in vitro* kinase assay was performed in reaction buffer (20 mM Tris-HCl pH 7.5, 5 mM MgCl_2_, 0.5 mM DTT, and 150 mM KCl, 5 mM ATP) and 250 ng of recombinant H3 as previously reported in a reaction volume of 40 μl during 45 min at 30°C (Martin-Doncel et al., 2019). H3T3ph was detected with a rabbit polyclonal antibody (Upstate-Millipore) (Salzano et al., 2015; Moura et al., 2018; Marcos et al., 2020).

### Antibodies

The primary and secondary antibodies used in this work are listed in Table 1. The monoclonal antibody 1B5 (anti-VRK1) detects the activated VRK1 under native conditions such as in immunofluorescence (Sanz-Garcia et al., 2012; Salzano et al., 2015; Monsalve et al., 2016; Campillo-Marcos and Lazo, 2019). In western blots using denatured proteins, the antibody does not discriminate between active and inactive forms.

### Immunoblots

After SDS-PAGE electrophoresis, proteins were transferred to PVDF Immobilon-P membranes (Millipore). PVDF membranes were activated in methanol (Sigma Aldrich) for 2 min. Then, gel cassettes were submerged in transfer buffer (25 mM Tris-HCl, 19.2 mM glycine and 10–20% methanol). Transfer was done at 90 V for 90 min in the cold. Membranes were blocked in 5% non-fat dried milk or BSA, diluted in TBS-T (25 mM Tris-HCl (pH 8.0), 50 mM NaCl, 2.5 mM KCl, and 0.1% Tween-20) in milli-Q H_2_O r 1 h at room temperature. Afterward, membranes were washed 3 times in TBS-T, followed by the incubation with the primary antibody in 1% BSA in TBS-T (Table 1) for 1–2 h at RT, or overnight at 4°C. Membranes were washed 3 times with TBS-T, followed by incubation with the secondary antibody (Table 1) at 1:10,000 dilution in 1% BSA in TBS-T in the dark. Membranes were washed 3 times in TBS-T and scanned using the Odyssey Infrared Imaging System (LI-COR Biosciences) to detect fluorescence. Membrane images were quantified using Quantity One software (Bio-Rad).

### Flow Cytometry

In order to study apoptosis, we used a commercial kit containing annexin V and 7-AAD (7-Amino-Actinomycin D) purchased from Immunostep (ref.: ANXVKB-100T). Annexin V is a Ca^+2^-dependent phospholipid binding protein which binds to phosphatidylserine when is translocated from the inner to the outer leaflet of the plasma membrane during apoptosis. Cells were centrifugated (1,200 rpm, 5 min) and the pellet was washed twice with PBS (1,200 rpm, 5 min). The pellet was resuspended in binding buffer containing annexin V (prepared according to the manufacturer) for 15 min in agitation in the dark. Finally, annexin V was detected by flow cytometry using Accuri C6, BD. Data was analyzed with Accuri C6 software.

### Statistics

All analysis were performed using IBM SPSS 25 and 26 software. Statistical significance was analyzed by non-parametric tests (Mann–Whitney *U*–test) (Bremer and Doerge, 2009; Pollard et al., 2019).

### Reagents

Temozolomide was from SelleckChem. Olaparib from LC Laboratories (Woburn, MA, United States). All other reagents were from Merck-Sigma-Aldrich.

## Results

### Temozolomide and Olaparib Cooperate in Sensitizing Glioblastoma Cells to DNA Damage

**FIGURE 1:**
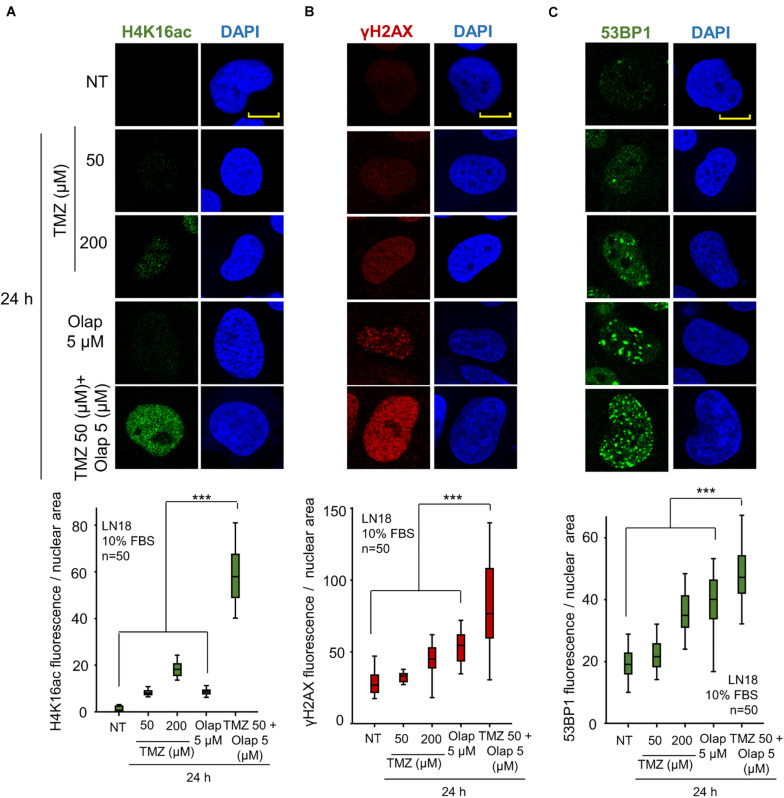
Effect of the combination of TMZ and olaparib on H4K16 acetylation levels, γH2AX and 53BP1 foci formation in response to DNA damage in LN-18 glioblastoma cells. **(A)** Effect of TMZ 50 and 200 μM, olaparib 5 μM and the combination of TMZ 50 and olaparib 5 μM on H4K16ac shown by immunofluorescence (IF). **(B)** Effect of TMZ 50 and 200 μM, olaparib 5 μM and the combination of TMZ 50 and olaparib 5 μM on γH2AX shown by IF. **(C)** Effect of TMZ 50 and 200 μM, olaparib 5 μM and the combination of TMZ 50 and olaparib 5 μM on 53BP1 shown by IF. Scale bar = 10 μm. ***p < 0.001. The quantification of fluorescence levels per nuclear area from fifty cells in triplicate is shown at the bottom. NT: no treatment. Scale bar = 10 μm. ***p < 0.001. Field images shown in Supplementary Figure 1.

Initially, we determined whether TMZ and olaparib could have a cooperative effect on the initial local relaxation of chromatin. This effect can be detected by the acetylation of histones, which subsequently can sensitize cells to DNA damage. The effect of these two inhibitors was determined on three sequential steps of the DDR, in two GBM cell lines, LN-18 and LN-229. First, the acetylation of histone H4 in K16 (H4K16ac); next, the early response to DNA damage, which was detected by the formation of γH2AX foci; and the activation of the NHEJ pathway detected by the formation of 53BP1 foci (Mirman and de Lange, 2020; Zhao et al., 2020). VRK1 directly phosphorylates VRK1 in Ser139 in H2AX (γH2AX) (Salzano et al., 2015), and 53BP1 in response to DNA damage. For this aim, GBM cells were treated with two doses of TMZ (50 and 200 μM) and olaparib (5 μM), and the combination of their lower doses (TMZ 50 μM and olaparib 5 μM) for 24 h. The initial chromatin relaxation after DNA damage was detected by H4K16ac (Figure 1A), which is followed by the accumulation of γH2AX (Figure 1B), as well as the formation of 53BP1 foci on damaged DNA (Figure 1C). TMZ and olaparib by themselves have little effect on H4K16ac and γH2AX foci, and a minor effect on 53BP1 foci (Figure 1). However, when using the combination of TMZ and olaparib at lower doses, a significant increase of H4K16ac, γH2AX, and 53BP1 foci in LN-18 (Figure 1 and Supplementary Figure 1) and LN-229 cells (Supplementary Figure 2) was observed. The combination of TMZ and olaparib led to a reduction in drug doses by 75% of TMZ causing a higher DNA damage response than with each single treatment at a higher concentration. Therefore, GBM cells show a higher DDR, reflecting the accumulation of DNA damage, when treated with a combination of TMZ and olaparib at lower doses.

### H4K16 Acetylation Induced by TMZ and Olaparib Is Impaired by VRK1 Depletion

**FIGURE 2:**
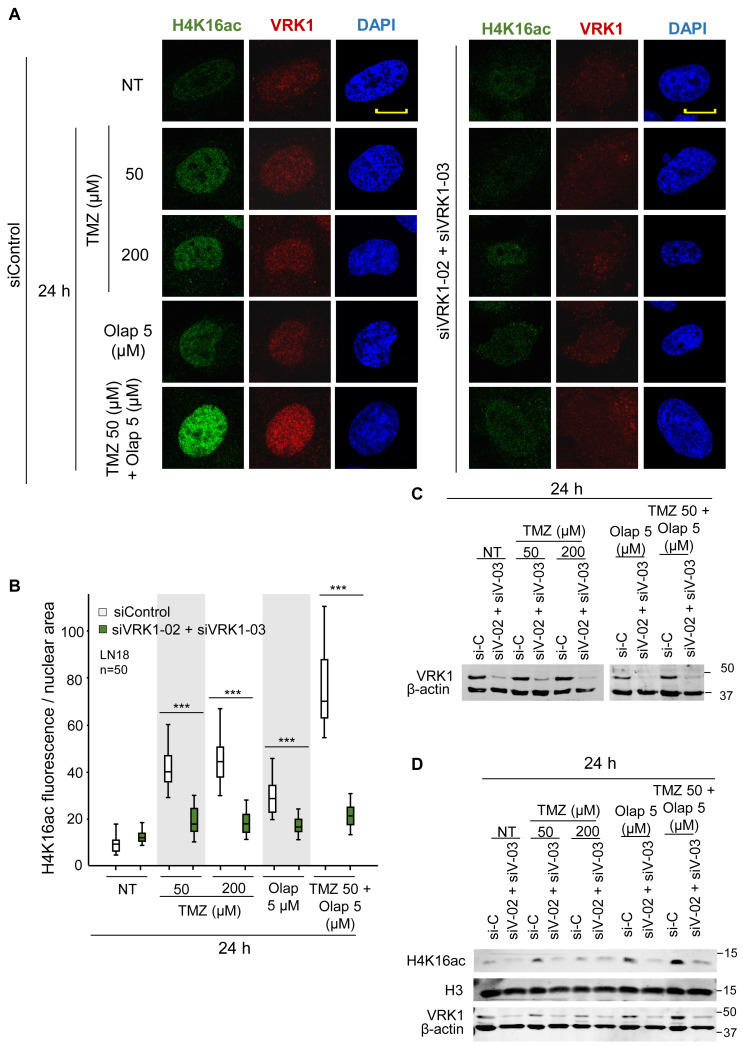
Effect of VRK1 depletion on H4K16 acetylation (H4K16ac) induced by TMZ and olaparib in LN-18 cells. **(A)** Left. Effect of siControl on H4K16ac induced by TMZ, olaparib and their combination. Right. Effect of the combination of siVRK1-02 and siVRK1-03 on H4K16ac induced by TMZ, olaparib and their combination. **(B)** Quantification of the effect of VRK1 depletion on H4K16ac per cellular area. Fifty cells in triplicate experiments were quantified. Field images are shown in Supplementary Figure 3. Scale bar = 10 μm. ***p < 0.001. **(C)** Western blot showing the effect of VRK1 knockdown. β-actin was used as load control. NT: no treatment. **(D)** Effect of the combination of TMZ and olaparib and VRK1 knock-down on H4K16ac detected in WB.

The acetylation of lysine 16 of histone 4 (H4K16ac) is an epigenetic modification that opens chromatin, in order to become accessible for the DNA repair machinery (Murr et al., 2006; Dhar et al., 2017). Induction of DNA damage causes a local relaxation of chromatin that is associated with histone H4K16 acetylation (Murr et al., 2006; Ito, 2007). The loss of H4K16ac is associated with defective DNA repair (Dhar et al., 2017). Moreover, Tip60/KAT5, which is directly phosphorylated and activated by VRK1 in response to DNA damage regulating H4K16 acetylation (Garcia-Gonzalez et al., 2020). Therefore, we studied the effect of VRK1 knockdown on H4K16ac in LN-18 and LN-229 cells that were treated with TMZ and olaparib. In these GBM cells, VRK1 was depleted and treated with TMZ 50 and 200 μM, olaparib 5 μM, and the combination of TMZ 50 and olaparib 5 μM for 24 h to determine their effect on chromatin relaxation associated to H4K16ac changes (Figure 2). The level of H4K16ac significantly decreased in VRK1-depleted cells after TMZ and olaparib combination treatment in LN-18 (Figure 2) and LN-229 cells (Supplementary Figure 4). These results suggest that VRK1 has a role in very early phases of DDR, being crucial for chromatin remodeling before the repair.

### Loss of VRK1 Impairs γH2AX Foci Induced by the Combination of TMZ and Olaparib

**FIGURE 3:**
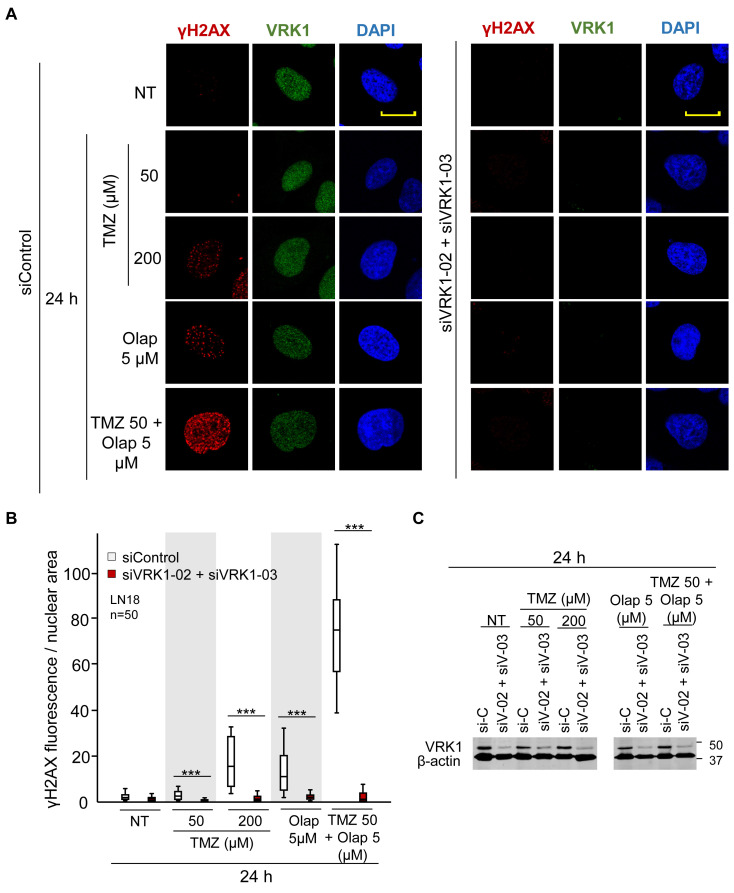
Effect of VRK1 knockdown on γH2AX foci formation after TMZ and olaparib treatments in LN-18 cells. **(A)** Left. Effect of siControl on γH2AX foci formation induced by TMZ, olaparib and their combination. Right. Effect of the combination of siVRK1-02 and siVRK1-03 on γH2AX foci formation induced by TMZ, olaparib and their combination. **(B)** Quantification of the effect of VRK1 depletion on γH2AX fluorescence per nuclear area. Fifty cells per experiment in triplicate were quantified. NT: no treatment. Scale bar = 10 μm. ***p < 0.001. Field images shown in Supplementary Figure 5. **(C)** Western blot showing the effect of VRK1 knockdown. β-actin was used as load control.

H2AX is directly phosphorylated in Ser139 (γH2AX) by VRK1 in response to ionizing radiation (Salzano et al., 2015), and is required for the accumulation of γH2AX foci to protect the damaged region (Fernandez-Capetillo et al., 2004; Bonner et al., 2008). Hence, next we studied whether VRK1 is also involved in DDR at other levels, besides chromatin remodeling in response to TMZ and olaparib treatments. Thus, we analyzed the effect of VRK1 depletion on the formation of γH2AX foci after treatments with TMZ, olaparib, or their combination. Cells were treated with TMZ (50 and 200 μM), olaparib (5 μM), and their combination at the lower doses (TMZ 50 and olaparib 5 μM) for 24 h, and the DDR was analyzed using γH2AX as an early marker. Depletion of VRK1 caused a significant decrease in the formation of γH2AX foci in response to TMZ and olaparib treatments, mainly when combined, in both LN-18 (Figure 3 and Supplementary Figure 5) and LN-229 cells (Supplementary Figure 6). These results indicate that VRK1 is involved in the DDR but not only in chromatin remodeling immediately after the damage, but also in sensing the lesions, and is acting at different levels of the sequential DDR steps.

### VRK1 Depletion Impairs H4K20me2 Accumulation and 53BP1 Recruitment to Damage Sites Induced by TMZ and Olaparib

**FIGURE 4:**
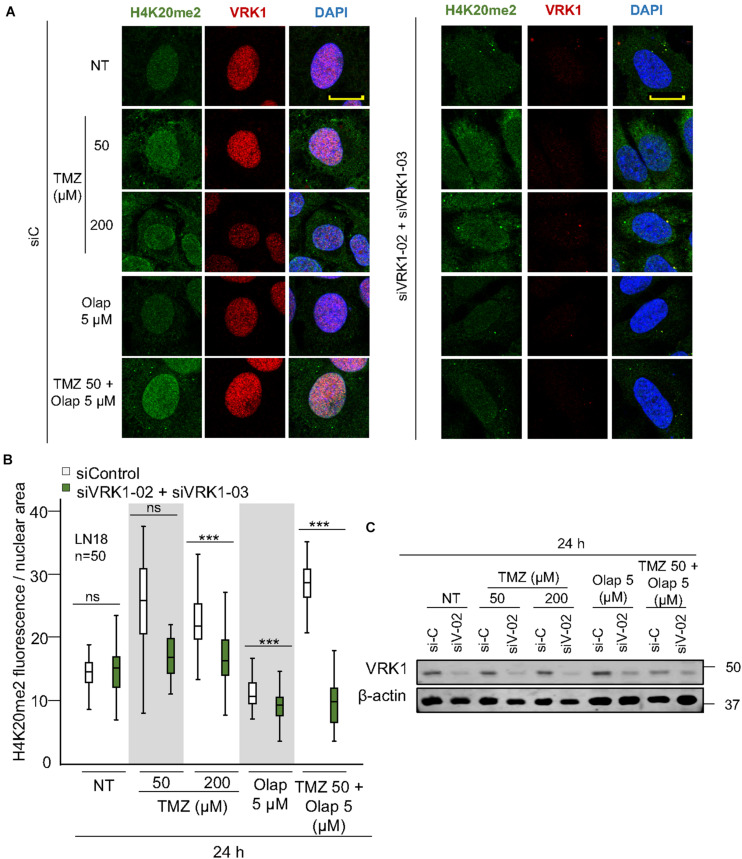
VRK1 knockdown impairs H4K20me2 after TMZ and olaparib treatments. **(A)** Left. Effect of siControl on H4K20me2 induced by TMZ, olaparib and their combination. Right. Effect of the combination of siVRK1-02 and siVRK1-03 on H4K20me2 induced by TMZ, olaparib and their combination. **(B)** Quantification of the effect of VRK1 depletion on H4K20me2 fluorescence per nuclear area. Fifty cells per condition in triplicate were quantified. Field images shown in Supplementary Figure 7. NT: no treatement. Scale bar = 10 μm. ***p < 0.001. **(C)** Western blot showing the effect of VRK1. β-actin was used as load control.

**FIGURE 5:**
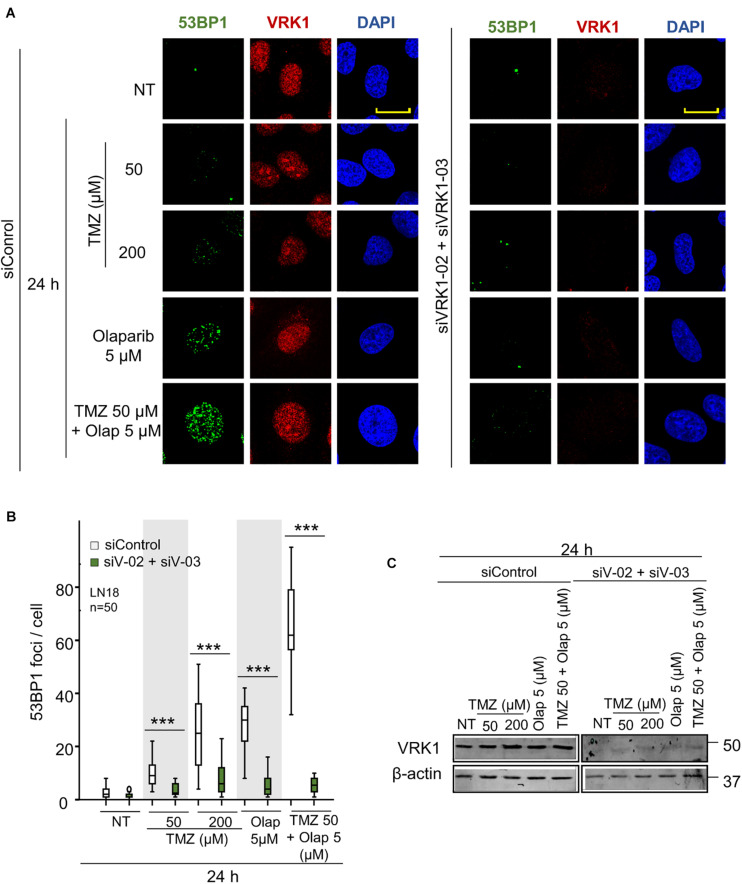
VRK1 knockdown prevents 53BP1 foci formation after TMZ and olaparib treatments in LN-18. **(A)** Left. Effect of siControl on 53BP1 foci formation induced by TMZ, olaparib and their combination. Right. Effect of the combination of siVRK1-02 and siVRK1-03 on 53BP1 foci formation induced by TMZ, olaparib and their combination. **(B)** Quantification of the effect of VRK1 depletion on 53BP1 fluorescence per nuclear area. Fifty cells per condition in triplicate were quantified. Field images shown in Supplementary Figure 9. NT: no treatment. Scale bar = 10 μm. ***p < 0.001. **(C)** Western blot showing the effect of VRK1. β-actin was used as load control.

NHEJ is the main repair pathway of DSB (Pannunzio et al., 2018; Zhao et al., 2020). An intermediate step in the NHEJ pathway, is mediated by the formation of 53BP1 foci at DNA damage sites (Sanz-Garcia et al., 2012), which requires the accumulation of H4K20me2 (Jacquet et al., 2016; Guo et al., 2018; Lou et al., 2020). Therefore, we studied the effect of VRK1 depletion on the accumulation of H4K20me2, and the formation 53BP1 foci induced by TMZ and olaparib. LN-18 and LN-229 cells were treated with TMZ 50 and 200 μM, olaparib 5 μM, and their combination at lower doses (TMZ 50 and olaparib 5 μM). VRK1 depletion resulted in a loss of H4K20me2 (Figure 4 and Supplementary Figure 7), an epigenetic modification required for 53BP1 foci assembly. Next, we studied the formation of 53BP1 foci, which acts later by protecting DNA ends in the DDR (Zhao et al., 2020). We observed a significant reduction in 53BP1 foci caused by VRK1 depletion in cells treated with either TMZ, olaparib or their combination in LN-18 cells (Figure 5 and Supplementary Figure 9). These results suggest that VRK1 is involved in the DDR at different levels and is necessary for a correct DDR in intermediate stages. Similar results were obtained in LN-229 cells (Supplementary Figures 8, 10).

### Temozolomide and Olaparib Cause the Accumulation of Free DNA 5’-Ends in VRK1 Depleted Cells

**FIGURE 6:**
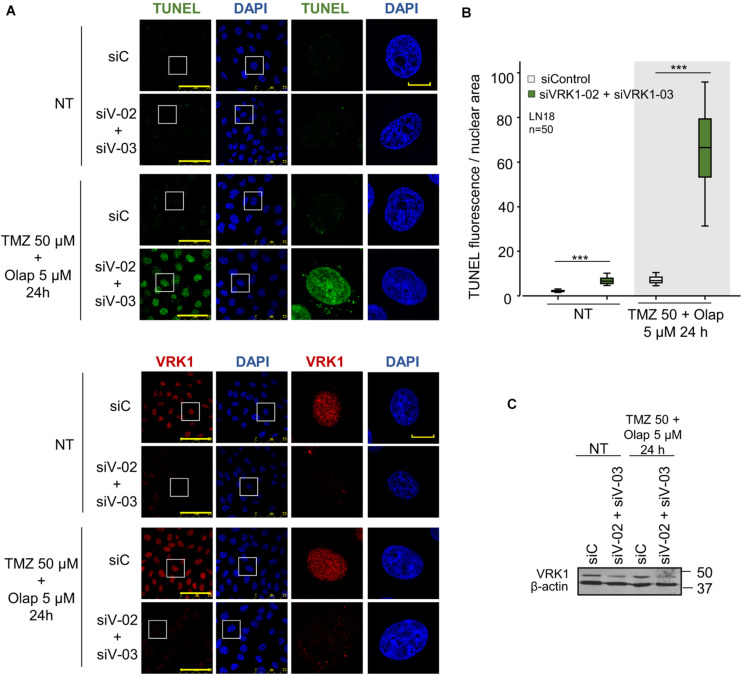
Effect of VRK1 knockdown on DNA damage induced by TMZ and olaparib treatments in LN-18. **(A)** Effect of siControl and the combination of siVRK1-02 and siVRK1-03 on DNA damage induced by TMZ, olaparib and the combination of both drugs. Free DNA ends resulting from DNA damage were detected in TUNEL assays. **(B)** Quantification of the effect of VRK1 depletion on DNA damage. Fifty cells per condition were quantified. Scale bar = 15 μm. ***p < 0.001. **(C)** Western blot showing the effect of VRK1 knockdown. β-actin was used as load control. NT: no treatment.

VRK1 regulates the response to DNA damage caused by ionizing radiation and doxorubicin (Sanz-Garcia et al., 2012; Monsalve et al., 2016; Campillo-Marcos and Lazo, 2019). Therefore, we tested whether VRK1 depletion could have an effect on the level of DNA damage caused by TMZ and olaparib. The accumulation of unrepaired DNA damage is an indicator of cell death. For this aim, VRK1 was depleted in LN-18 and LN-229 cells followed by treatment with the combination of TMZ and olaparib. In these cells, DNA damage was detected by TUNEL assays that label free DNA-ends in broken strands. The combination of TMZ and olaparib cause a low level of DNA damage, detected by a minor increase in free DNA-ends (Figure 6). However, VRK1 depletion caused a very significant increase in the labeling of free-DNA ends in both LN-18 (Figure 6) and LN-229 cells (Supplementary Figure 11) treated with the TMZ and olaparib combination. This result indicates that VRK1 depletion sensitizes GBM cells to DNA damaging agents, such as the combination of TMZ and olaparib, and facilitates the accumulation of DNA damage.

### Temozolomide and Olaparib Combined With VRK1 Depletion Cause a Loss of Cell Viability and an Increase in Glioblastoma Cell Death

**FIGURE 7:**
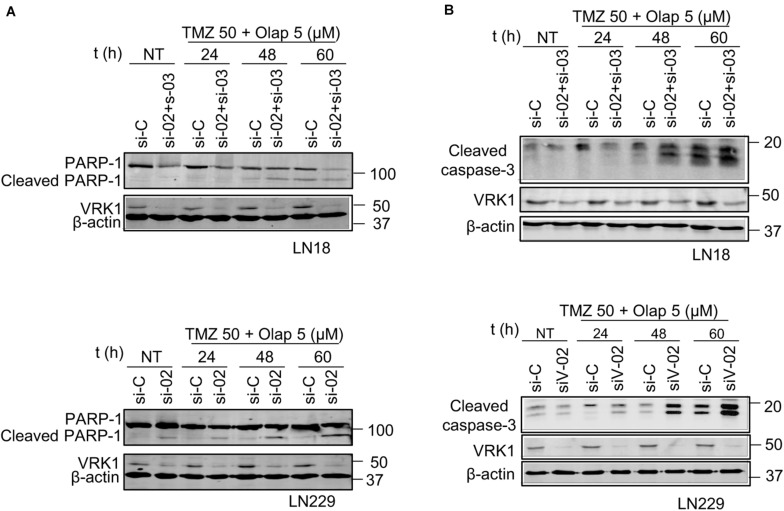
Effect of VRK1 knockdown on cell death induced by TMZ and olaparib treatments in LN-18 and LN-229. **(A)** Effect of the combination of VRK1 depletion by siVRK1-02 and siVRK1-03 on PARP1 after TMZ, olaparib and the combination of both drugs. Western blot showing total protein levels of full-length PARP1 (116 kDa) and cleaved PARP1 (86 kDa) in LN-18 (top) and LN-229 (bottom). **(B)** Effect of the combination of VRK1 depletion by siVRK1-02 and siVRK1-03 on caspase-3 processing. Western blot showing total protein levels of cleaved caspase-3 in LN-18 (top) and LN-229 (bottom). Cleaved PARP1 and cleaved caspase-3 were used as markers of caspase activation. β-actin was used as load control.

The previous data indicate that the DNA damage caused by TMZ and olaparib could not be repaired in the absence of VRK1. Therefore, the most likely consequence will be the facilitation of tumor cell death. To test this possibility, we determined the processing of PARP-1, as an apoptosis biomarker, which reflects the activation of caspases that cleave PARP-1 and caspase-3. In GBM cells, treated with the combination of TMZ 50 (μM) and olaparib 5 (μM) at different time points, it was detected a decrease of full-length PARP-1 and an increase in cleaved PARP-1, and cleaved caspase-3, in both LN-18 (Figure 7A) and LN-229 (Figure 7B) GBM cells after drug treatments. These results indicate that VRK1 depletion leads to an increase in cell death, which was confirmed by the detection of an increase in the Annexin V + population in flow cytometry assays (Supplementary Figure 12). Taken together, our results indicate that VRK1 depletion is impairing the DDR triggered by TMZ, olaparib or their combination in GBM cells. This inability of having a functional DDR is leading to an increase of tumor cell cytotoxicity and death. Thus, we propose the VRK1 kinase as a good candidate target for novel therapeutic strategies based on synthetic lethality in glioblastomas.

## Discussion

Glioblastomas are tumors with poor prognosis and limited treatment options that require the identification of novel therapeutic strategies. In this context, strategies based on synthetic lethality are proving useful in different types of tumors (Lord et al., 2008; Williamson et al., 2010; Bouwman and Jonkers, 2012; Leichman et al., 2016; Bourton et al., 2017; Sizemore et al., 2018; Visnes et al., 2018; Campillo-Marcos and Lazo, 2019). These strategies are based on the inactivation of alternative DDR pathways, required for tumor progression, to promote tumor cell death. This strategy was developed in tumors that already have mutations in specific DDR genes, such as BRCA1 (Bourton et al., 2017), BRCA2 (Fong et al., 2009), or WRN (Chan et al., 2019), which were treated with additional drugs that target proteins in different DDR pathways, such as olaparib. An alternative approach, in the absence of a mutation in a DDR gene, is to target two different DDR pathways with specific inhibitors, which can cause a pharmacological DDR deficiency, and thus facilitate tumor cell death.

High levels of VRK1 have been associated with resistance to treatment in glioblastomas (Varghese et al., 2016). This effect is a consequence of the role of VRK1 in facilitating the activation of p53 (Vega et al., 2004; Lopez-Sanchez et al., 2014) and in DNA damage responses (Sanz-Garcia et al., 2012; Salzano et al., 2015; Monsalve et al., 2016; Campillo-Marcos and Lazo, 2018), which makes this protein, and its function, a potential therapeutic target. Several CRISPR/Cas9 screenings have identified *VRK1* as a driver gene (Kiessling et al., 2016; Behan et al., 2019) and as potential candidate for therapeutic development (Jacoby et al., 2015), which is consistent with its identification as a kinase that can be used in synthetic lethality strategies (Huang et al., 2020).

In this report, we have studied the effect that VRK1 depletion has in glioblastoma cells, based on the observation that VRK1 is a chromatin kinase which regulates several steps in the DDR (Sanz-Garcia et al., 2012; Salzano et al., 2014; Salzano et al., 2015; Monsalve et al., 2016) and impairs the NHEJ pathway (Sanz-Garcia et al., 2012). We have shown that VRK1 depletion sensitizes tumor cells to two types of pharmacological treatments, temozolomide, a drug used in glioblastoma, and olaparib, an inhibitor of PARP-1 that is in use for the treatment of tumors with BRCA1 mutations (Tewari et al., 2015; Robson et al., 2017; Golan et al., 2019). Combination strategies can contribute to minimizing the development of resistance to cancer treatments based on TMZ (Zhang et al., 2012; Perazzoli et al., 2015; Kaina and Christmann, 2019; Higuchi et al., 2020), by facilitating a reduction of the drug dose in those tumors in which there is a different DDR pathway, which can be simultaneously targeted.

The role of VRK1 as a chromatin kinase that controls chromatin reorganization and DDR can be a potentially suitable therapeutic alternative. VRK1 depletion led to an increase in sensitivity to DNA-damage inducing drugs, such as TMZ or olaparib and their combination. Thus, VRK1 depletion facilitates a reduction of dose to achieve a similar effect on the impairment of DDR. This hypersensitization effects, a consequence of VRK1 depletion, is higher than that of TMZ or olaparib, by themselves or combined.

Moreover, this effect is independent of the MGMT or p53 status of the glioblastoma cells. This synthetic lethality of VRK1 depletion was detected when used in combination with DNA damage treatments, such as ionizing radiation or doxorubicin, which permitted a reduction of their doses (Campillo-Marcos and Lazo, 2019).

Depletion of VRK1 in combination with TMZ and olaparib in glioblastoma cells causes an increase in DNA damage at lower doses, which results in tumor cell death. Thus, targeting VRK1 can become a therapeutic option when specific VRK1 inhibitors are developed. The catalytic domain of VRK1 has some unique structural differences (Manning et al., 2002). These differences make it feasible to develop specific inhibitors. The study of the thermal shift of kinases induced by binding to inhibitors detected that VRK1 is specific, and does not change with drugs targeting all families of the human kinome (Fedorov et al., 2007a). Therefore, VRK1 has a very low promiscuity index, which might permit development of highly specific inhibitors (Fedorov et al.,2007a,b; Eswaran and Knapp, 2010). This observation was confirmed in kinase assays using inhibitors targeting different families of the human kinome, in which none inhibited VRK1 (Vazquez-Cedeira et al., 2011). However, there are no inhibitors available for clinical use that target VRK1. The synthetic lethality of VRK1 depletion was also detected when used in combination with ionizing radiation or doxorubicin. VRK1 depletion led to a significant reduction in the dose needed to achieve a similar effect (Campillo-Marcos and Lazo, 2018, 2019; Garcia-Gonzalez et al., 2020). Recently, a pyrimidine-based inhibitor has shown high affinity and specificity for the VRK1 kinase (Serafim et al., 2019), which can be a candidate for future drug development.

## Conclusion

Depletion of the VRK1 chromatin kinase in glioblastoma cells enhances the DNA damage caused by temozolomide and olaparib treatments. The development of specific kinase inhibitors targeting the chromatin kinase VRK1, thus altering DNA damage responses, can be an alternative pharmacological option in the development of new combinatorial therapeutic strategies that can reduce drug toxicity and improve patient quality of life and survival, particularly in tumors that have no alterations in DDR pathways.

## Data Availability Statement

The original contributions presented in the study are included in the article/Supplementary Material, further inquiries can be directed to the corresponding author/s.

## Author Contributions

EN-C designed and performed experiments, analyzed data, and wrote a draft of the manuscript. PL designed experiments, coordinated the project, analyzed data, and wrote the manuscript. Both authors contributed to the final manuscript.

## Conflict of Interest

The authors declare that the research was conducted in the absence of any commercial or financial relationships that could be construed as a potential conflict of interest.

## Abbreviations

BER: Base excision repair
BRCA1/2: BRCA1/2 DNA Repair Associated
DDR: DNA-damage response
DSB: Double strand break
GBM: glioblastoma
IF: Immunofluorescence
KAT5: Lysine acetyl transferase 5
MGMT: O-6-methylguanine-DNA methyltransferase
NHEJ: non-homologous end joining
Olap: olaparib
PARP: Poly (ADP-Ribose) Polymerase
TMZ: temozolomide
TUNEL: Terminal deoxynucleotidyl transferase dUTP nick end labeling
VRK1: vaccinia-related kinase 1
WRN: WRN RecQ Like Helicase
53BP1: p53 binding protein-1.
