# Supplementary material for: VRK1 Depletion Facilitates the Synthetic Lethality of Temozolomide and Olaparib in Glioblastoma Cells
Source: Front Cell Dev Biol. 2021 Jun 14;9:683038. doi: 10.3389/fcell.2021.683038 (PMC8237761; doi:10.3389/fcell.2021.683038)
Supplement: Supplementary file 1 [file Data_Sheet_1.PDF]

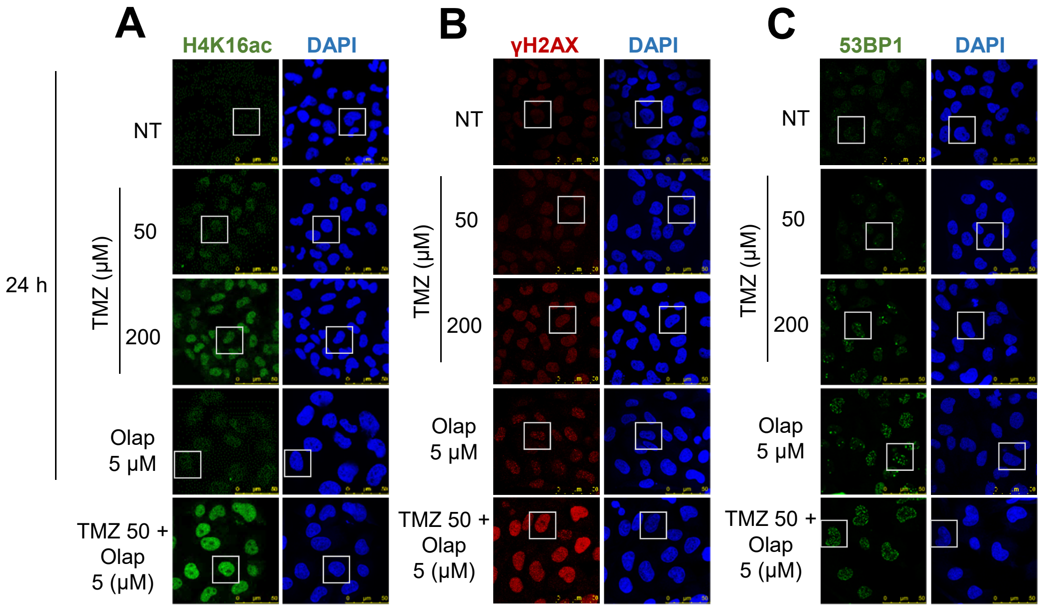

Figure S1. Effect of the combination of TMZ and olaparib on H4K16 acetylation levels, and γH2AX and 53BP1 foci formation in response to DNA damage in LN-18 glioblastoma cells. **A.** Effect of TMZ 50 and 200 µM, olaparib 5 µM and the combination of TMZ 50 and olaparib 5 µM on H4K16ac. **B.** Effect of TMZ 50 and 200 µM, olaparib 5 µM and the combination of TMZ 50 and olaparib 5 µM on γH2AX shown by IF. **C.** Effect of TMZ 50 and 200 µM, olaparib 5 µM and the combination of TMZ 50 and olaparib 5 µM on 53BP1. Field images from Figure 1. Squares indicate the cells shown in Figure 1. NT: no treatment.
