# Supplementary material for: VRK1 Depletion Facilitates the Synthetic Lethality of Temozolomide and Olaparib in Glioblastoma Cells
Source: Front Cell Dev Biol. 2021 Jun 14;9:683038. doi: 10.3389/fcell.2021.683038 (PMC8237761; doi:10.3389/fcell.2021.683038)
Supplement: Supplementary file 2 [file Data_Sheet_2.PDF]

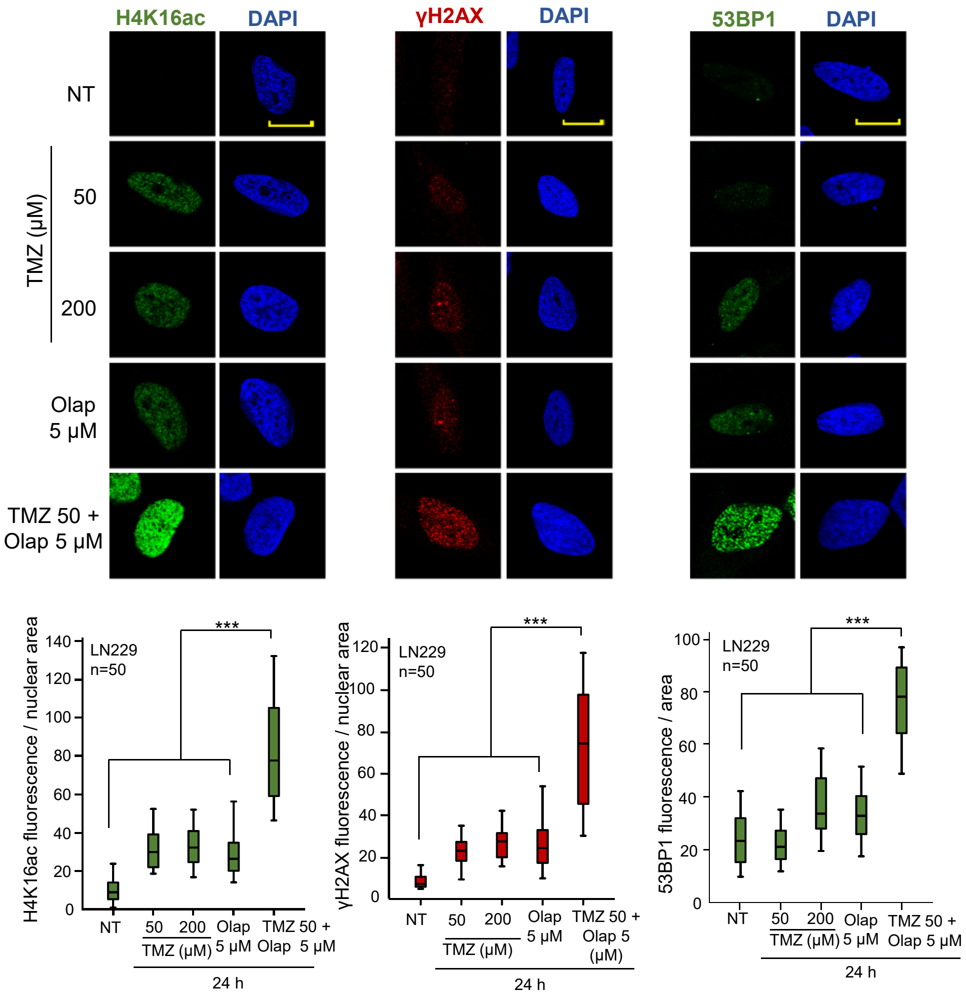

Figure S2. Effect of the combination of TMZ and olaparib on H4K16ac acetylation levels, γH2AX and 53BP1 foci formation in response to DNA damage in LN-229 glioblastoma cell line. Top. Effect of TMZ 50 and 200 μM, olaparib 5 μM and the combination of TMZ 50 and olaparib 5 μM on H4K16ac, γH2AX and 53BP1 shown by IF. **Bottom**. Quantification of H4K16ac, γH2AX and 53BP1 fluorescence levels per nuclear area. Fifty cells per condition were quantified in triplicate experiments. Scale bar= 15 μm. . *** *P* < 0.001.
