# Supplementary material for: VRK1 Depletion Facilitates the Synthetic Lethality of Temozolomide and Olaparib in Glioblastoma Cells
Source: Front Cell Dev Biol. 2021 Jun 14;9:683038. doi: 10.3389/fcell.2021.683038 (PMC8237761; doi:10.3389/fcell.2021.683038)
Supplement: Supplementary file 4 [file Data_Sheet_4.PDF]

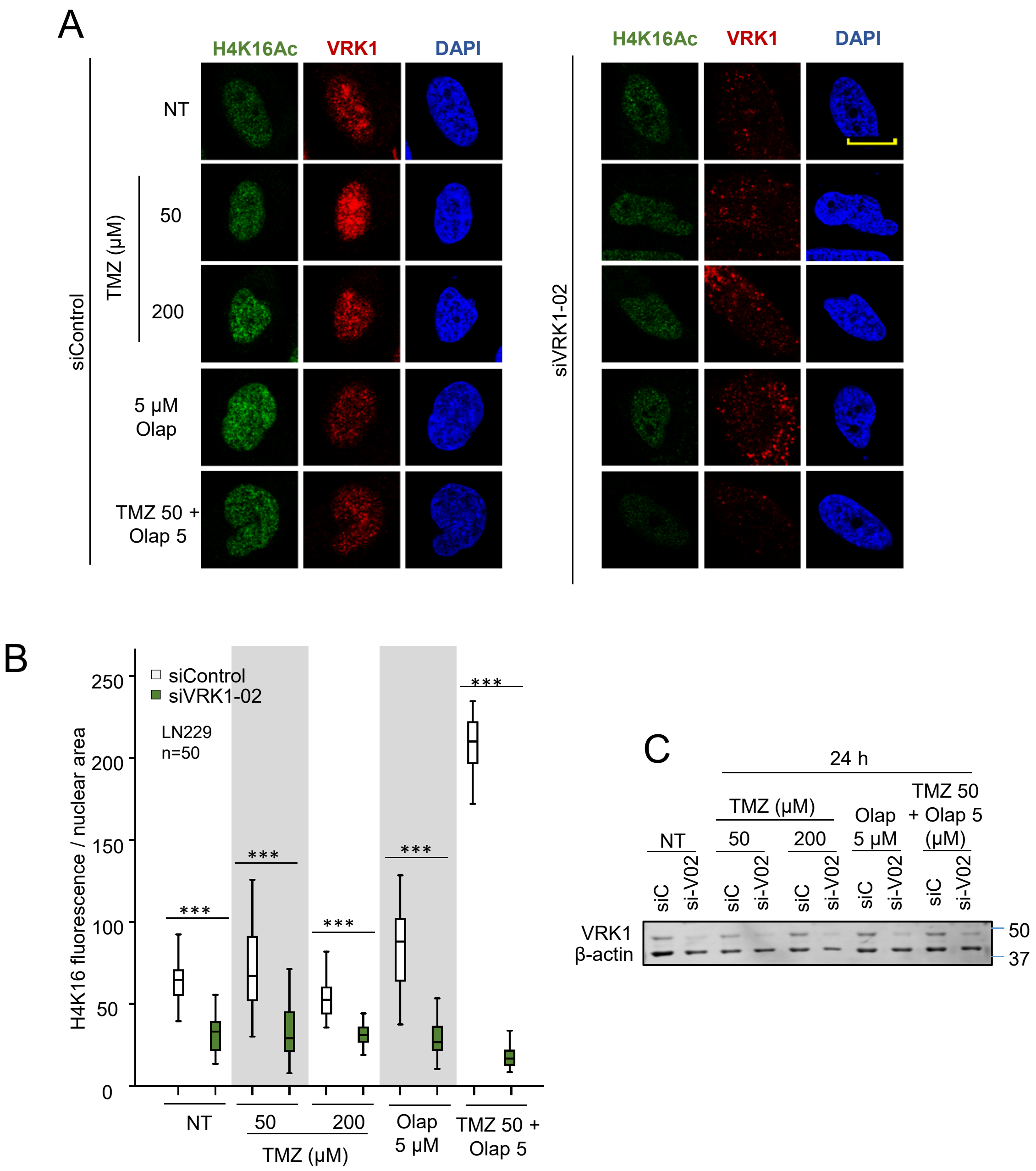

Figure S4. Effect of VRK1 knockdown on H4K16 acetylation induced by TMZ and olaparib in LN-229 cells. **A**. Left. Effect of siControl on H4K16ac induced by TMZ, olaparib and their combination. **A.** Right. Effect of siVRK1-02 on H4K16ac induced by TMZ, olaparib and their combination. **B**. Quantification of the effect of VRK1 depletion on H4K16ac per nuclear area. NT: no treatment. Fifty cells per condition were quantified. ***p < 0.001 **C**. Western blot showing the effect of VRK1 depletion β-actin was used as load control. Scale bar= 10 μm.
