# Supplementary material for: VRK1 Depletion Facilitates the Synthetic Lethality of Temozolomide and Olaparib in Glioblastoma Cells
Source: Front Cell Dev Biol. 2021 Jun 14;9:683038. doi: 10.3389/fcell.2021.683038 (PMC8237761; doi:10.3389/fcell.2021.683038)
Supplement: Supplementary file 5 [file Data_Sheet_5.PDF]

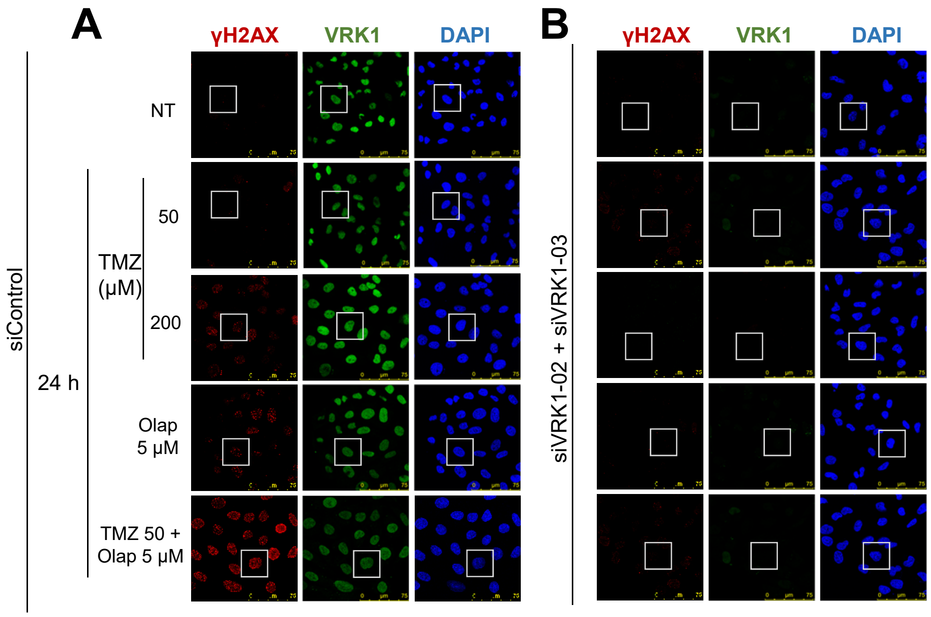


**Figure S5.** Effect of VRK1 knockdown on γH2AX foci formation induced by TMZ and olaparib in LN-18. **A**. Effect of siControl on γH2AX induced by TMZ, olaparib and the combination of both drugs shown by IF. **B.** Effect of the combination of siVRK1-02 and siVRK1-03 on γH2AX after TMZ, olaparib and their combination shown by IF. Field images from Figure 3. Squares indicate the cells shown in Figure 3.
