# Supplementary material for: VRK1 Depletion Facilitates the Synthetic Lethality of Temozolomide and Olaparib in Glioblastoma Cells
Source: Front Cell Dev Biol. 2021 Jun 14;9:683038. doi: 10.3389/fcell.2021.683038 (PMC8237761; doi:10.3389/fcell.2021.683038)
Supplement: Supplementary file 6 [file Data_Sheet_6.PDF]

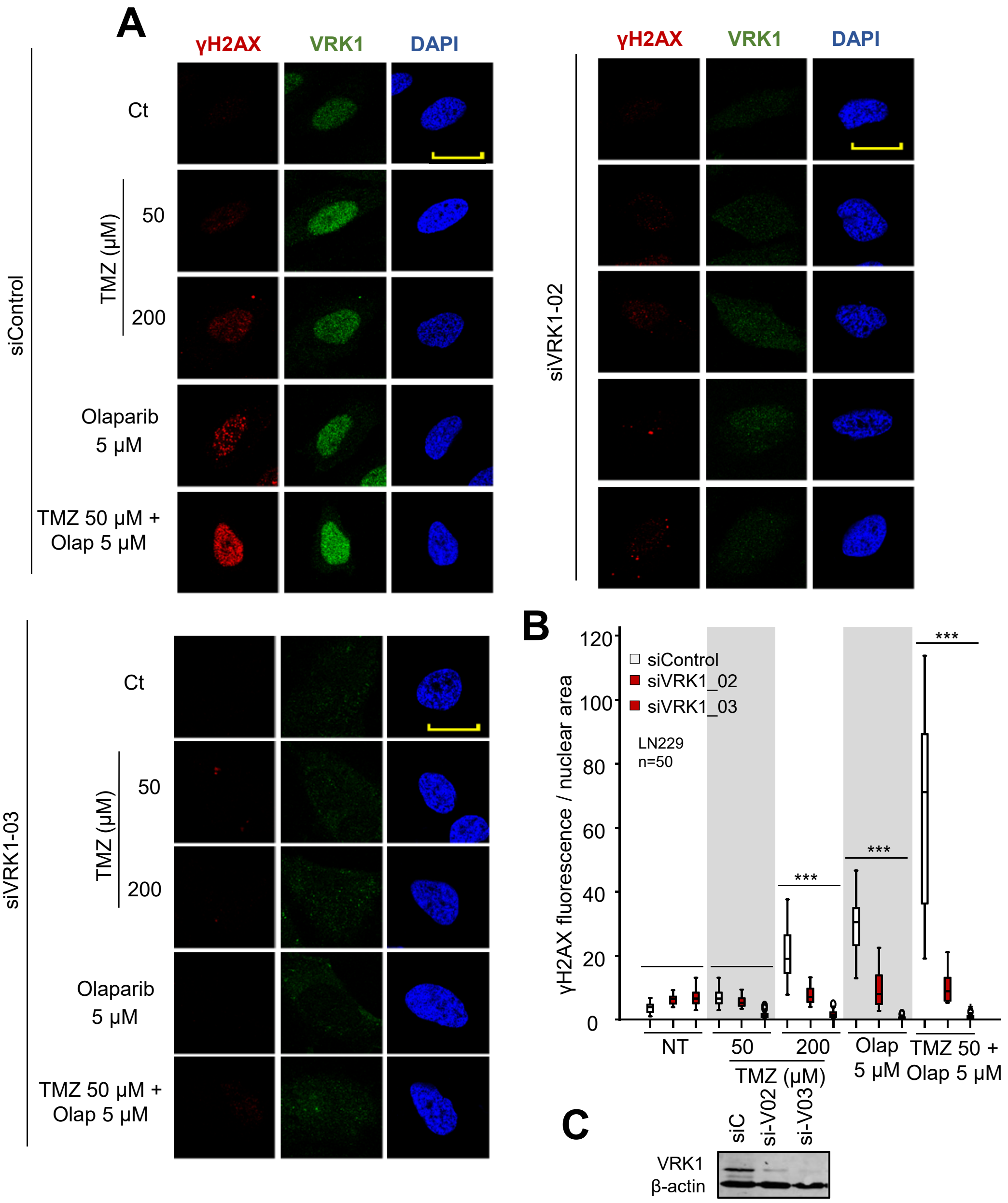

Figure S6. Effect of VRK1 knockdown on γH2AX foci formation after TMZ and olaparib treatments in LN-229. **A**. Left. Effect of siControl on γH2AX foci formation induced by TMZ, olaparib and their combination. A. Right and down. Effect of siVRK1-02 and siVRK1-03 on γH2AX foci formation induced by TMZ, olaparib and their combination. Scale bar= 15 μm. **B**. Quantification of the effect of VRK1 depletion on γH2AX fluorescence per nuclear area. Fifty cells per condition were quantified. ***$P < 0.001$. **C**. Western blot showing the effect of VRK1 depletion in cell extracts. β-actin was used as load control.
