# Supplementary material for: VRK1 Depletion Facilitates the Synthetic Lethality of Temozolomide and Olaparib in Glioblastoma Cells
Source: Front Cell Dev Biol. 2021 Jun 14;9:683038. doi: 10.3389/fcell.2021.683038 (PMC8237761; doi:10.3389/fcell.2021.683038)
Supplement: Supplementary file 7 [file Data_Sheet_7.pdf]

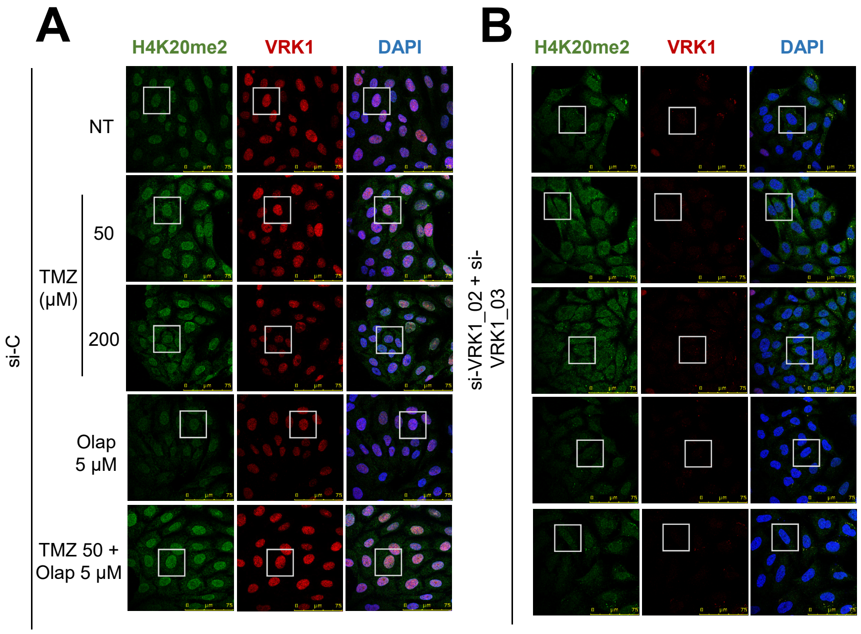

Figure S7. Effect of VRK1 knockdown on H4K20me2 induced by TMZ and olaparib in LN-18. **A.** Effect of siControl on H4K20me2c induced by TMZ, olaparib and the combination of both drugs shown by IF. **B.** Effect of siVRK1-02 and siVRK1-03 on H4K20me2 induced by TMZ, olaparib and their combination shown by IF. NT: no treatment. Field images from Figure 4. Squares indicate the cells shown in Figure 4.
