# Supplementary material for: VRK1 Depletion Facilitates the Synthetic Lethality of Temozolomide and Olaparib in Glioblastoma Cells
Source: Front Cell Dev Biol. 2021 Jun 14;9:683038. doi: 10.3389/fcell.2021.683038 (PMC8237761; doi:10.3389/fcell.2021.683038)
Supplement: Supplementary file 9 [file Data_Sheet_9.PDF]

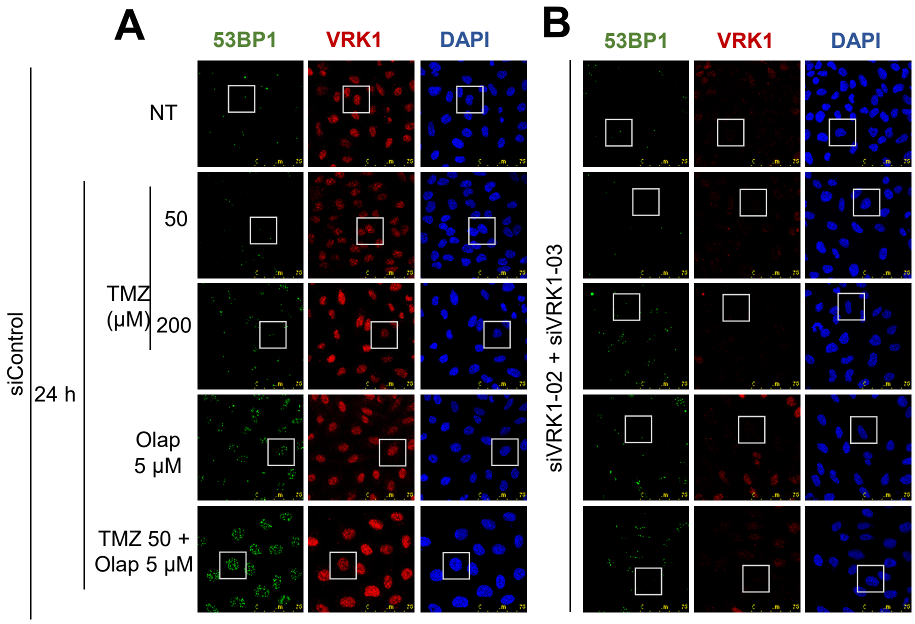

Figure S9. Effect of VRK1 knockdown on 53BP1 foci formation induced by TMZ and olaparib in LN-18. **A**. Effect of siControl on 53BP1 induced by TMZ, olaparib and the combination of both drugs shown by immunofluorescence. **B.** Effect of the combination of siVRK1-02 and siVRK1-03 on 53BP1 after TMZ, olaparib and their combination shown by immunofluorescence. Field images from Figure 4. Squares indicate the cells shown in Figure 4.
