# Supplementary material for: VRK1 Depletion Facilitates the Synthetic Lethality of Temozolomide and Olaparib in Glioblastoma Cells
Source: Front Cell Dev Biol. 2021 Jun 14;9:683038. doi: 10.3389/fcell.2021.683038 (PMC8237761; doi:10.3389/fcell.2021.683038)
Supplement: Supplementary file 10 [file Data_Sheet_10.PDF]

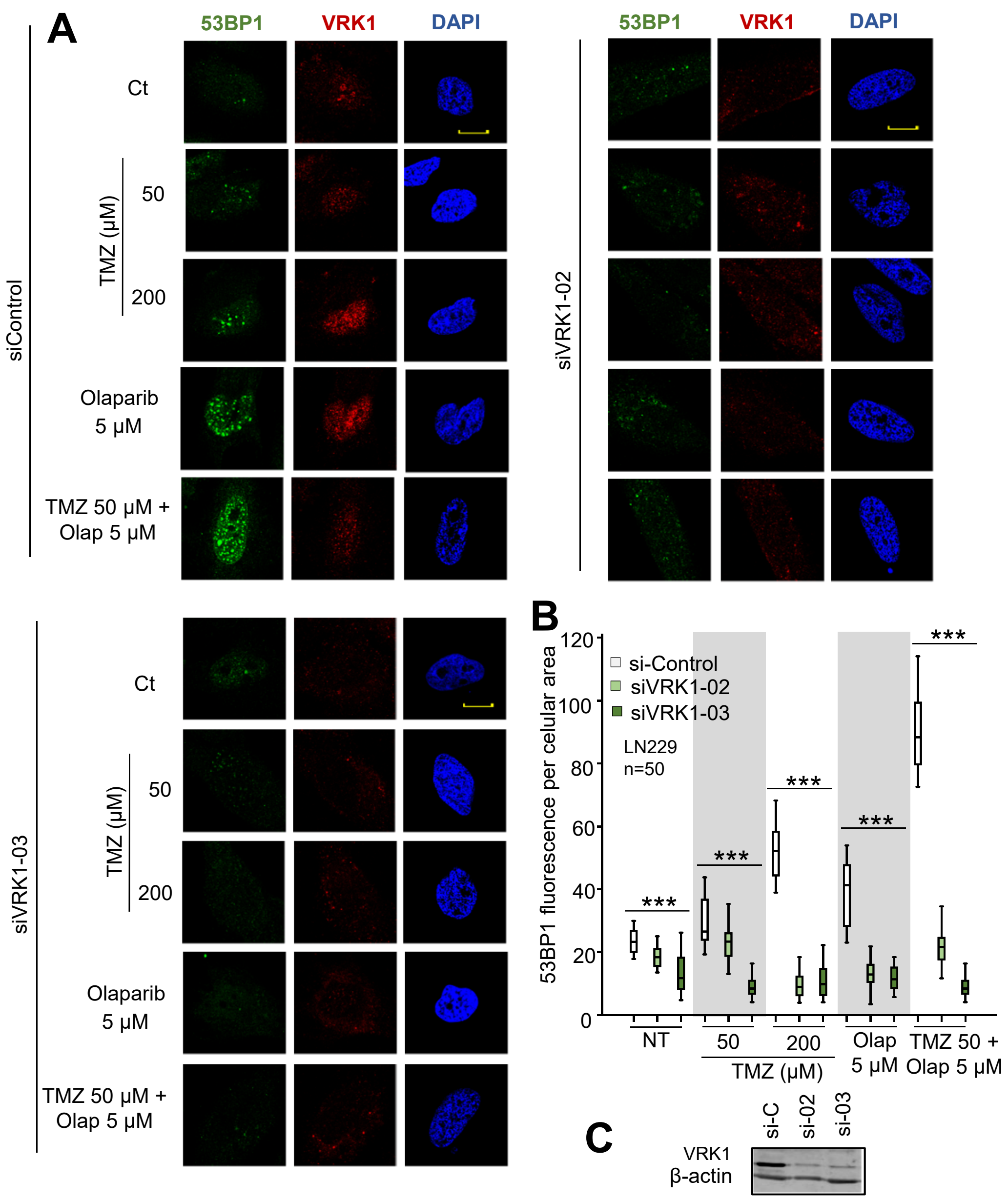

Figure S10. Effect of VRK1 knockdown on 53BP1 foci formation after TMZ and olaparib treatments in LN-229**. A**. Left. Effect of siControl on 53BP1 foci formation induced by TMZ, olaparib and their combination. **A**. Right and down. Effect of the combination of siVRK1-02 and siVRK1-03 on 53BP1 foci formation induced by TMZ, olaparib and their combination. **B**. Quantification of the effect of VRK1 depletion on 53BP1 fluorescence per nuclear area. Fifty cells were used for quantification. ***P < 0.001. Scale bar= 15 µm. **C**. Western blot showing the effect of VRK1 in cell lysates. β-actin was used as load control.
