# Supplementary material for: VRK1 Depletion Facilitates the Synthetic Lethality of Temozolomide and Olaparib in Glioblastoma Cells
Source: Front Cell Dev Biol. 2021 Jun 14;9:683038. doi: 10.3389/fcell.2021.683038 (PMC8237761; doi:10.3389/fcell.2021.683038)
Supplement: Supplementary file 11 [file Data_Sheet_11.PDF]

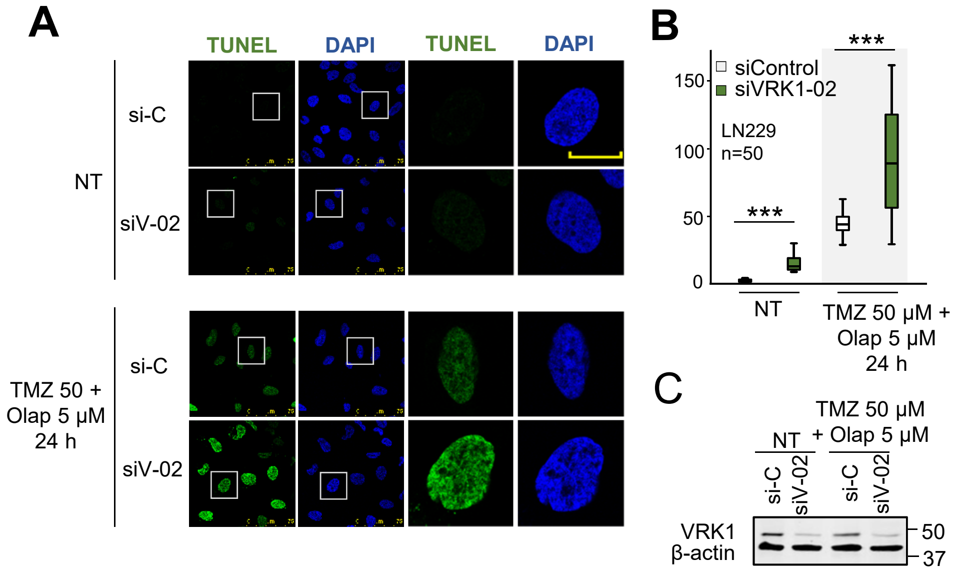

Figure S11. Effect of VRK1 knockdown on DNA damage induced by TMZ and olaparib treatments in LN-229. **A**. Effect of siControl, siVRK1-02 and siVRK1-03 on DNA damage induced by TMZ, olaparib and the combination of both drugs. Nick DNA ends resulting from DNA damage labeled and detected using a TUNEL assays. NT: no treatment. Scale bar= 15 µm. ***P < 0.001. **B**. Quantification of the effect of VRK1 depletion on DNA damage. Fifty cells per condition were quantified. **C**. Western blot showing the effect of VRK1 depletion on total population. β-actin was used as control.
