# Supplementary material for: VRK1 Depletion Facilitates the Synthetic Lethality of Temozolomide and Olaparib in Glioblastoma Cells
Source: Front Cell Dev Biol. 2021 Jun 14;9:683038. doi: 10.3389/fcell.2021.683038 (PMC8237761; doi:10.3389/fcell.2021.683038)
Supplement: Supplementary file 12 [file Data_Sheet_12.PDF]

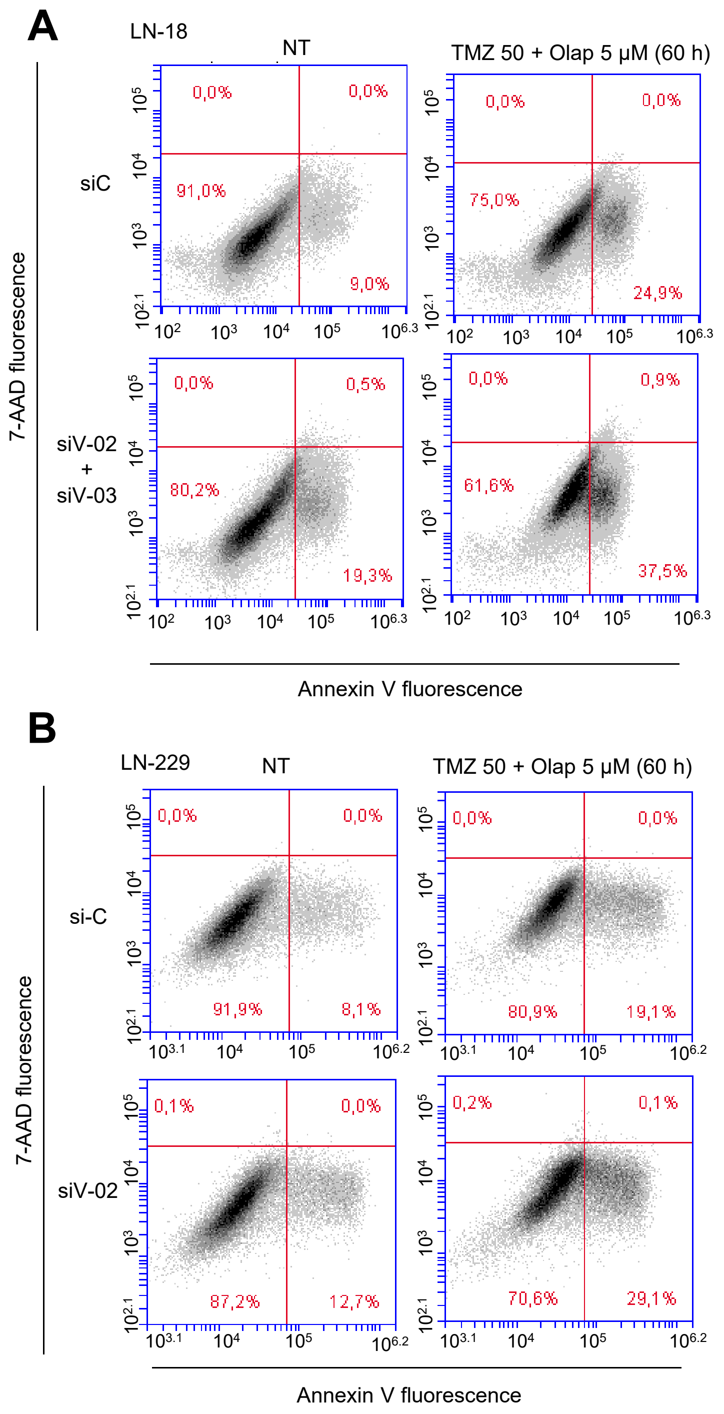

Figure S12. Effect of VRK1 knock-down on cell death induced by TMZ and olaparib in LN-18 (A) and LN-229 (B) cells. Effect of siControl (siC) and the combination of siVRK1-02 (siV-02) and siVRK1-03 (siV-03) on cell death induced by the combination of TMZ and olaparib in LN-18 and LN-229 cells. Annexin V and 7-AAD were used as biomarkers and fluorescence was analyzed by flow cytometry. NT: no treatment..
